# Taurine activates GABAergic networks in the neocortex of immature mice

**DOI:** 10.3389/fncel.2014.00026

**Published:** 2014-02-04

**Authors:** Bogdan A. Sava, Rongqing Chen, Haiyan Sun, Heiko J. Luhmann, Werner Kilb

**Affiliations:** Institute of Physiology, University Medical Center MainzMainz, Germany

**Keywords:** GABA_A_ receptor, glycine receptor, development, neocortex, GABAergic excitation, interneuron, cortical plate, subplate

## Abstract

Although it has been suggested that taurine is the main endogenous neurotransmitter acting on glycine receptors, the implications of glycine receptor-mediated taurine actions on immature neocortical networks have not been addressed yet. To investigate the influence of taurine on the excitability of neuronal networks in the immature neocortex, we performed whole-cell patch-clamp recordings from visually identified pyramidal neurons and interneurons in coronal slices from C57Bl/6 and GAD67-green fluorescent protein (GFP) transgenic mice (postnatal days 2–4). In 46% of the pyramidal neurons bath-application of taurine at concentrations ≥ 300 μM significantly enhanced the frequency of postsynaptic currents (PSCs) by 744.3 ± 93.8% (*n* = 120 cells). This taurine-induced increase of PSC frequency was abolished by 0.2 μM tetrodotoxin (TTX), 1 μM strychnine or 3 μM gabazine, but was unaffected by the glutamatergic antagonists 6-cyano-7-nitroquinoxaline-2,3-dione (CNQX) and (±) R(-)-3-(2-carboxypiperazine-4-yl)-propyl-1-phosphonic acid (CPP), suggesting that taurine specifically activates GABAergic network activity projecting to pyramidal neurons. Cell-attached recordings revealed that taurine enhanced the frequency of action potentials (APs) in pyramidal neurons, indicating an excitatory action of the GABAergic PSCs. In order to identify the presynaptic targets of taurine we demonstrate that bath application of taurine induced in GAD67-GFP labeled interneurons an inward current that is mainly mediated by glycine receptors and can generate APs in these cells. We conclude from these results that taurine can enhance network excitability in the immature neocortex by selectively activating GABAergic interneurons via interactions with glycine receptors.

## Introduction

A variety of studies reported that ionotropic receptors for γ-amino butyric acid (GABA) are functionally expressed during pre- and early post-natal development (e.g., Laurie et al., 1992; Fritschy et al., 1994; Van Eden et al., 1995) and that such GABA_A_ receptors mediate depolarizing responses in immature hippocampal (Ben-Ari et al., 1989; Rivera et al., 1999; Sipila et al., 2005) and neocortical neurons (Owens et al., 1996; Achilles et al., 2007; Ben-Ari et al., 2012). GABA_A_ receptors are critically involved in several developmental steps (reviewed in Wang and Kriegstein, 2009), like regulating neuronal proliferation (LoTurco et al., 1995; Haydar et al., 2000), migration (Behar et al., 2000; Cuzon et al., 2006; Heck et al., 2007), neurite growth (Sernagor et al., 2010) and synaptic integration (Wang and Kriegstein, 2008). Most of these effects are mediated by tonic GABAergic activation of non-synaptic receptors (reviewed in Kilb et al., 2013). In addition to these non-synaptic processes, GABAergic synaptic inputs had been demonstrated in various neuronal populations of the neocortex during pre- and early post-natal stages (Owens et al., 1999; Kilb and Luhmann, 2001; Hanganu et al., 2002; Radnikow et al., 2002; Rheims et al., 2008). It has been suggested that GABAergic depolarizations provide the excitatory drive necessary for early network activity (Sipila et al., 2005; Ben-Ari, 2006, 2012), although in the neocortex early synchronous network oscillations are mainly independent of GABAergic signaling (Garaschuk et al., 2000; Allene et al., 2008). Such early network activity probably also plays an important role in the functional maturation of neocortical circuits (Ben-Ari, 2002; Spitzer, 2006; Hanganu-Opatz, 2010; Kilb et al., 2011).

On the other hand, no major anatomically disturbances of the perinatal neocortex and hippocampus were observed in the absence of GABA (Ji et al., 1999) or synaptic GABA release (Wojcik et al., 2006), suggesting that other intrinsic GABAergic agonists may act on GABA_A_ receptors during early development. Taurine, a partial agonist of GABA_A_ (Albrecht and Schousboe, 2005) and glycine receptors (Flint et al., 1998), is one possible candidate. Taurine can be released by a variety of stimuli in the adult nervous system (Saransaari and Oja, 1998; Oja and Saransaari, 2000). A non-vesicular release of taurine can be induced by electrical stimulation and in the presence of a hypoosmolar solution in the immature neocortex (Flint et al., 1998; Kilb et al., 2008). Taurine has been shown to be directly involved in the development of the central nervous system (CNS; Palackal et al., 1986; Sturman et al., 1994; Maar et al., 1995; Behar et al., 2001) and influences the shift from depolarizing to hyperpolarizing GABAergic responses (Inoue et al., 2012). In accordance with the depolarizing effect of GABA, taurine also depolarizes different neuronal populations of the immature neocortex via an activation of GABA_A_ and glycine receptors (Ito and Cherubini, 1991; Flint et al., 1998; Kilb et al., 2002, 2008). However, the effect of such depolarizing taurine responses on the network activity in the immature neocortex has to our knowledge not been investigated yet.

To analyze the influence of taurine on network activity in the immature neocortex we investigated the effect of taurine application on the spontaneous postsynaptic currents (PSCs) recorded in pyramidal neurons of the mouse neocortex at postnatal days (P) 2–4. These experiments revealed that taurine induced a massive increase in the frequency of GABAergic PSCs, which is suppressed by the glycinergic agonist strychnine and mediated an excitatory response in these cells. Recordings from genetically labeled GABAergic interneurons showed that taurine induced an inward current, mainly mediated by glycine receptors, and thereby generate action potentials (APs) in these cells. These findings suggest that in the early postnatal neocortex taurine can activate GABAergic networks via an interaction with glycine receptors on GABAergic interneurons and may thereby influence the maturation of neocortical network.

## Materials and Methods

### Preparation of the cortical slices

Animal handling was performed in accordance with EU directive 86/609/EEC for the use of animals in research and approved by the local ethical committee (Landesuntersuchungsanstalt RLP, Koblenz, Germany). All efforts were made to minimize the number of animals and their suffering. For electrophysiological experiments coronal slices of the neocortex were prepared from pups of C57Bl/6 or GAD67-GFP transgenic mice (Tamamaki et al., 2003) at P2–P4. Mice were deeply anesthetized by enflurane (Abbot, Wiesbaden, Germany). After opening of the skull, the brain was quickly removed and placed in ice-cold artificial cerebrospinal fluid (ACSF; composition given below). Whole-brain 400 μM thick coronal slices were cut on a vibratome (HR2, Sigmann Elektronik, Hüffenhardt, Germany) and cut along the midline to separate the two hemispheres. Only coronal slices that according to an atlas of the developing rat brain (Paxinos and Franklin, 2001) included the primary somatosensory cortex were transferred to an incubation-storage chamber. Slices were allowed to recover for at least 1 h before recording. For some experiments a reduced slice preparation was required. For that coronal slices with a thickness of 400 μM were placed under a binocular and trimmed with a scalpel blade to a width of approximately 2–3 mm. In some of these slices the subplate (SP) and white matter were identified by eye and the SP was removed with a scalpel blade.

### Electrophysiological Setup

The videomicroscopic setup consisted of an upright microscope (BW51WI, Olympus) with infrared differential interference contrast (IR-DIC) optics (Dodt and Zieglgänsberger, 1990), and a CCD-camera (C5405, Hamamatsu, Japan). The video image was contrast enhanced by a video processor (C 2400, Hamamatsu) and visualized on a video-monitor. For whole-cell and cell-attached patch-clamp experiments pipettes were made from borosilicate tubing (2.0 mm outside diameter, 1.16 mm inside diameter; Science Products, Hofheim, Germany) using a vertical puller (PP-83, Narishige, Tokyo, Japan) and filled with pipette solution (see below). The patch pipettes were connected to the headstage of a discontinuous voltage-clamp/current-clamp amplifier (SEC05L, NPI, Tamm, Germany). Signals were amplified, low-pass filtered at 3 kHz, visualized on an oscilloscope (HM507, Hameg Instruments), digitized on-line by an AD/DA-board (ITC-18, Heka, Lamprecht, Germany), recorded and processed with the software WINTIDA 5.0 (Heka), and stored on a personal computer. The slices were transferred into a submerged recording chamber, mounted on the fixed stage of the microscope and were superfused with ACSF at a rate of 1–2 ml/min. All experiments were performed at 30 ± 1°C maintained by a peltier-element based temperature controller (Luigs & Neumann, Ratingen, Germany).

### Electrophysiological procedures and analysis

Resting membrane potential (RMP) was measured directly after establishing whole-cell configuration. All potentials were corrected for a liquid junction potential of 9.1 mV. Input resistance and capacitance were determined from amplitude and rise kinetics of voltage deflections to 300 ms hyperpolarizing current pulses. AP amplitude was defined as voltage difference between firing threshold and peak depolarization and AP width was measured at half-maximal amplitude. PSCs were detected and analyzed in continuous voltage-clamp recordings lasting at least 180 s at a holding potential (*E*_h_) of −69.1 mV (except where noted), using the program MiniAnalysis 4.3.3 (Synaptosoft, Leonia, NJ, USA). Frequencies of APs and PSCs were determined during at least 3 min of the recording immediately before agonist application for control conditions, and during the complete period of agonist application for the agonist condition. Holding currents were determined during a *ca.* 1 min interval directly before agonist application for control conditions and during a similar interval in the maximal phase of the agonist response. Cells were considered as responsive to agonists, if the frequency of PSCs in the presence of agonists (calculated from inter-event intervals) was 4x larger than the standard deviation of the frequency during the control interval. For taurine concentrations ≤ 100 μM cells were only considered as non-responsive, if the cells showed a taurine-induced increase in PSC frequency at higher taurine doses. The reversal potential (*E*_Rev_) was calculated from the Goldman equation using Cl^-^ concentrations, estimated ${\mathrm{HCO}}_{3}^{-}$ concentrations and a relative bicarbonate permeability of 0.2 (Bormann et al., 1987). Cell-attached experiments were performed either under current-clamp conditions at a holding-potential of −60 mV or under bridge-mode condition with no holding-current applied. Cells were considered as responsive to taurine application if AP frequency increases by more than 50% and more than five APs occur. In most experiments agonists and antagonists were applied via the bathing-solution. For some pharmacological experiments taurine, glycine, or isoguvacine was applied focally to the soma of the investigated cells via a patch pipette for 2–100 ms with a pressure of 0.4 bar using a pressure application system (LHDA0533115H, Lee, Westbrook, CT, USA).

### Solutions and substances

ACSF contained (in mM) 124 NaCl, 26 NaHCO_3_, 1.25 NaH_2_PO_5_, 1.8 MgCl_2_, 1.6 CaCl_2_, 3 KCl, 20 glucose (equilibrated with 95% O_2_/5% CO_2_), osmolarity 333 mOsm (determined by a freezing point osmometer, Knauer, Berlin, Germany). Standard pipette solution for whole-cell recordings contained (in mM) 44 KCl, 80 K-gluconate, 1 CaCl_2_, 2 MgCl_2_, 11 ethylene glycol tetraacetic acid (EGTA), 10 4-(2-hydroxyethyl)-1-piperazineethanesulfonic acid (HEPES), 2 Na_2_-ATP, 0.5 Na-GTP, pH adjusted to 7.4 with KOH and osmolarity to 306 mOsm with sucrose. For low-Cl^-^ pipette solution, the KCl concentration was reduced to 2 mM and the K-Gluconate concentration raised to 124 mM. For cell-attached recordings the pipette solution contained (in mM) 126 K-Gluconate, 4 KCl, 1 CaCl_2_, 2 MgCl_2_, 11 EGTA, 10 HEPES, 2 Na_2_-ATP, 0.5 Na-GTP, pH adjusted to 7.4 with KOH and osmolarity to 306 mOsm with sucrose.

Taurine was dissolved directly in ACSF, gabazine (SR-95531), 6-cyano-7-nitroquinoxaline-2,3-dione (CNQX) or strychnine were used from a stock solution in dimethylsulfoxide (DMSO), tetrodotoxin (TTX) from a stock solution in citrate buffer and glycine or (±) R(-)-3-(2-carboxypiperazine-4-yl)-propyl-1-phosphonic acid (CPP) from an aqueous stock solution. TTX was purchased from RBI (Natic, MA), glycine and taurine from Tocris (Ballwin, MO), and all other substances from Sigma. The final DMSO concentration never exceeded 0.5%.

### Staining procedures

In all whole-cell experiments 0.5 % biocytin (Sigma, Taufkirchen, Germany) was added to the pipette solutions to label the recorded neurons. Slices were fixed in a 4% parafolmaldehyde solution immediately after recording for more than 24 h. After washing in phosphate buffer, slices were incubated in blocking solution (4% normal goat and 3% normal bovine serum, 0.5% Triton, 0.05% sodium azide in phosphate buffered saline (PBS)) for 2 h at RT. Subsequently, biocytin-labeled neurons were stained with Cy-3 conjugated streptavidin (Dianova, Hamburg, Germany) as described in detail before (Achilles et al., 2007). Since the green fluorescent protein (GFP) fluorescence was not stable during the biocytin-streptavidin reaction, these slices were subsequently counterstained for GFP by incubating the slices with a GFP antibody raised in rabbit (1:500, Invitrogen, Karlsruhe, Germany) overnight at RT followed by a donkey anti-rabbit IgG secondary antibody conjugated with Dylight 488 (1:100; Dianova). Slices were embedded in fluoromount (Sigma). Immunofluorescence was investigated with a Nipkow spinning disk confocal system (Visitech, Sunderland, UK) attached to a conventional fluorescence microscope (Olympus BX51 WI) equipped with water immersion objectives and a cooled CCD-camera (CoolSnap HQ, Roper Scientific) controlled by MetaMorph software (Universal Imaging, West Chester, USA). Green and red fluorescence was excited with the 488 nm and the 568 nm lines of a Kr/Ar laser (Laser Physics, Malpas, UK).

### Statistics

All values are expressed as mean ± standard error of the mean (SEM). For statistical analysis Student *t*-test, Mann-Whitney-*U*-test, ANOVA tests and Fishers Exact test were used (Systat 11, Systat Inc., Point Richmond, CA). Results were designated significant at a level of *p* < 0.05.

## Results

### Basic properties of the investigated cells

**Figure 1:**
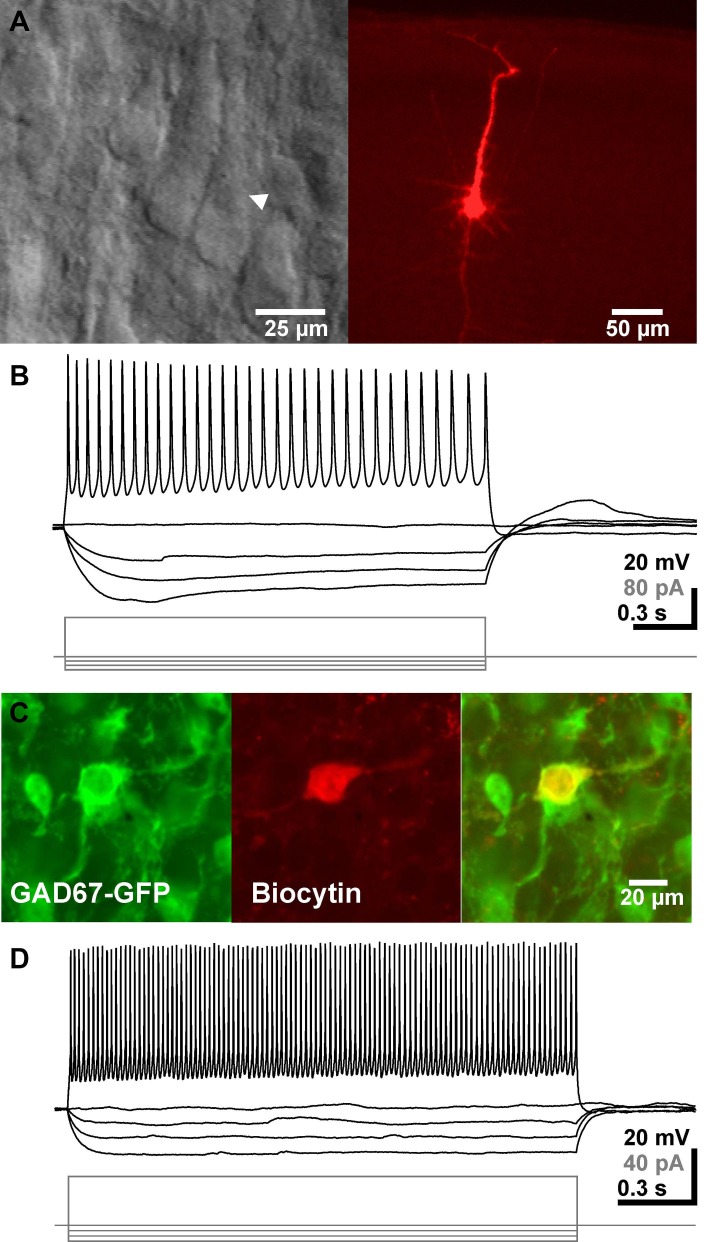
**Identification and basic electrophysiological properties of neocortical pyramidal neurons and GABAergic interneurons**. **(A)** Typical interference contrast image of a pyramidal neuron (arrowhead in left image) and the same neuron after biocytin-labeling and subsequent histochemical processing. **(B)** Voltage-traces of the neuron shown in **A** upon injection of de- and hyperpolarizing current pulses. **(C)** Confocal fluorescence images displaying GFP-positive interneurons (*left*), a biocytin-labeled recorded neuron (*middle*) and the merged picture (*right*). **(D)** Representative voltage traces of a GABAergic interneuron. Note the high firing frequency in this recording.

In total 530 pyramidal neurons from P2–P4 mice pups identified by their location in the neocortex and their pyramidal like appearance in IR-DIC image were investigated using whole-cell patch-clamp recordings (Figure 1A). These neurons had an average RMP of −68.9 ± 0.4 mV (*n* = 530), an input resistance of 1.7 ± 0.04 GΩ and an input capacity of 72.8 ± 2.5 pF. In 96% of all 450 neurons investigated for this aspect APs with an average amplitude of 54.8 ± 0.4 mV (*n* = 428) and duration of 4.4 ± 0.08 ms could be evoked upon a depolarization that crosses the AP-threshold of −41.7 ± 0.3 mV (Figure 1B).

In addition, 38 GFP^+^ GABAergic interneurons were recorded under whole-cell conditions (Figure 1C). These GABAergic interneurons had an average RMP of −64.6 ± 1.8 mV (*n* = 38), an input resistance of 3.3 ± 0.3 GΩ and an input capacity of 37.2 ± 5.0 pF. Injection of a depolarizing current evoked in these neurons APs with an amplitude of 37 ± 1.8 mV (*n* = 35), a duration of 2.9 ± 0.3 ms and an AP-threshold of −45.1 ± 0.9 mV (Figure 1D). Upon persistent injection of depolarizing currents these cell could discharge with a maximal frequency of 44.2 ± 2.8 Hz (*n* = 30), with a considerable heterogeneity.

### Focal taurinergic responses

**Figure 2:**
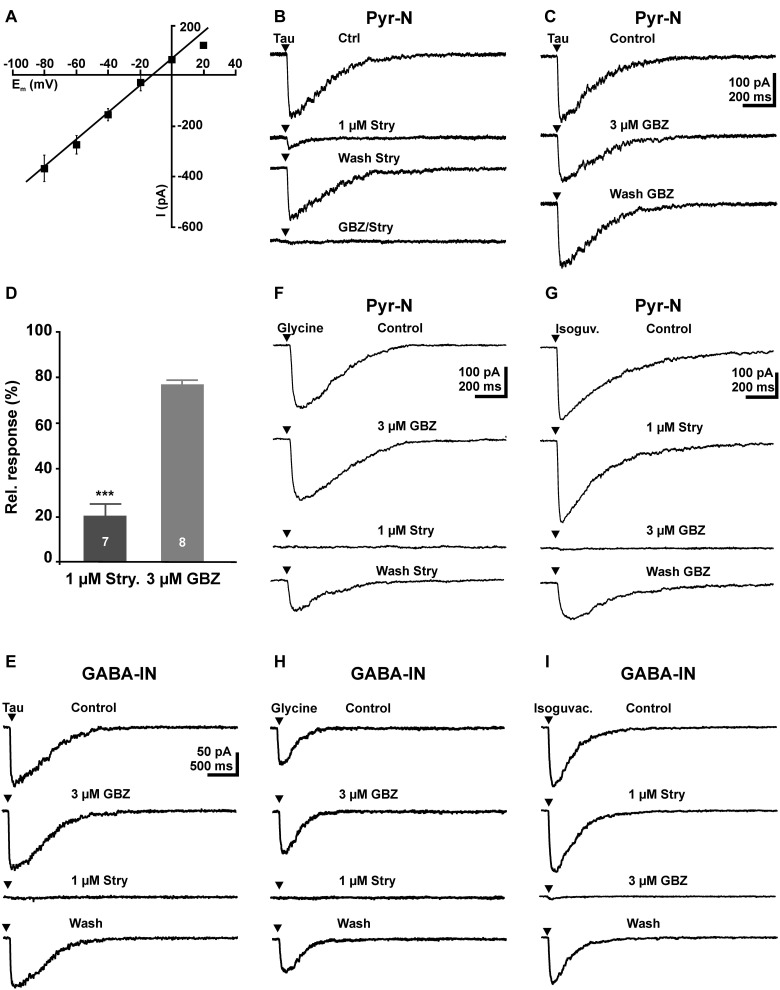
**Activation of GABA_A_ and glycine receptors by focal application of 5 mM taurine in pyramidal neurons and GABAergic interneurons. (A)** Current-voltage (I–V) plot of the inward current after focal application of 5 mM taurine at different voltages. Note the linear I–V relation and that the current reversed close to the estimated reversal potential. **(B)** Inward currents elicited by a 5 ms application of 5 mM taurine (Tau) are reduced in the presence of 1 μM strychnine (Stry). After a partially washout of strychnine, combined application of 3 μM gabazine (GBZ) and 1 μM strychnine completely suppressed the taurine-induced current. **(C)** Inward currents elicited by 5 mM taurine are slightly reduced in the presence of 3 μM GBZ. **(D)** Statistical analysis of these experiments. Bars represent mean ± SEM of maximal inward current amplitudes. **(E)** In GABAergic interneurons, focal application of 5 mM taurine induced an inward current that is unaffected by 3 μM GBZ and completely blocked in the presence of 1 μM strychnine. **(F)** In pyramidal neurons focal application of 100 μM glycine induced an inward current that was unaffected in the presence of 3 μM GBZ and completely blocked in the presence of strychnine. **(G)** In pyramidal neurons focal application of 100 μM isoguvacine induced an inward current that was unaffected by strychnine and completely blocked in the presence of GBZ. **(H)** In GABAergic interneurons focal application of 100 μM glycine induced an inward current that was unaffected by GBZ, but completely blocked in the presence of strychnine. **(I)** In GABAergic interneurons focal application of 100 μM isoguvacine induced an inward current that was completely blocked by GBZ, but unaffected in the presence of strychnine.

To investigate the pharmacology of postsynaptic taurine responses, we first applied 5 mM taurine directly to the soma of the recorded pyramidal cell. Focal taurine application (2–5 ms) induced in pyramidal neurons an inward current of −260.4 ± 31.9 pA (*n* = 12), which reversed at −22.3 ± 1.5 mV (*n* = 10, Figure 2A), in accordance with the Cl^-^ concentration of 50 mM in the pipette solution. Bath application of 1 μM strychnine significantly (*p* < 0.001) reduced the amplitude of taurine-induced currents by 80 ± 5.2% (*n* = 7, Figures 2B, D). Bath application of 3 μM gabazine reduced the amplitude of taurine-induced currents by 22 ± 2.5% (*n* = 8, Figures 2C, D), and the combined application of 1 μM strychnine and 3 μM gabazine completely blocked taurine induced currents (*n* = 6, Figure 2B). Interestingly, in GABAergic interneurons the taurine-induced inward current of 154.8 ± 22 pA (*n* = 10) was completely blocked by 1 μM strychnine (*n* = 9), while 3 μM gabazine (*n* = 8) had no effect (99.3 ± 6.6%; Figure 2E). In summary, these results suggest that the inward currents induced by high taurine concentrations were in pyramidal cells carried by both GABA_A_ and glycine receptors, with the GABA_A_ receptors mediating approximately 20% and glycine receptors approximately 80% of the responses, while in interneurons this current was exclusively mediated by glycine receptors.

As it had been shown that the pharmacological properties of glycine and GABA_A_ receptors change during development (Aguayo et al., 2004; Carlson and Yeh, 2011), we next performed control experiments to analyze the efficacy of strychnine and gabazine in immature pyramidal cells. In these experiments focal application of 100 μM glycine induced an inward current of 180.2 ± 42.5 pA (*n* = 9) that was completely blocked by 1 μM strychnine (*n* = 6), while 3 μM gabazine (*n* = 7) had no effect (103 ± 7.4%, *n* = 7, Figure 2F). Focal application of the GABAergic agonist isoguvacine (100 μM) induced an inward current of 382.3 ± 37.9 pA (*n* = 7) that was completely blocked by 3 μM gabazine (*n* = 6), while 1 μM strychnine had no effect (99 ± 1.8%, *n* = 4, Figure 2G). Similar results were obtained in interneurons. Focal application of 100 μM glycine induced in GABAergic interneurons a current of 91.4 ± 21.2 pA (*n* = 7) that was completely blocked by 1 μM strychnine (*n* = 7), whereas 3 μM gabazine had no effect 106.2 ± 5.3% (Figure 2H). Focal application of 100 μM isoguvacine induced in GABAergic interneurons a current of 131.6 ± 40.7 pA (*n* = 7) that was completely blocked by 3 μM gabazine (*n* = 7), whereas 1 μM strychnine had no effect (96.3 ± 3.8%; Figure 2I). These experiments demonstrate that 1 μM strychnine and 3 μM gabazine can be used as specific antagonists for glycine and GABA_A_ receptors, respectively.

### Effect of taurine on spontaneous synaptic currents

**Figure 3:**
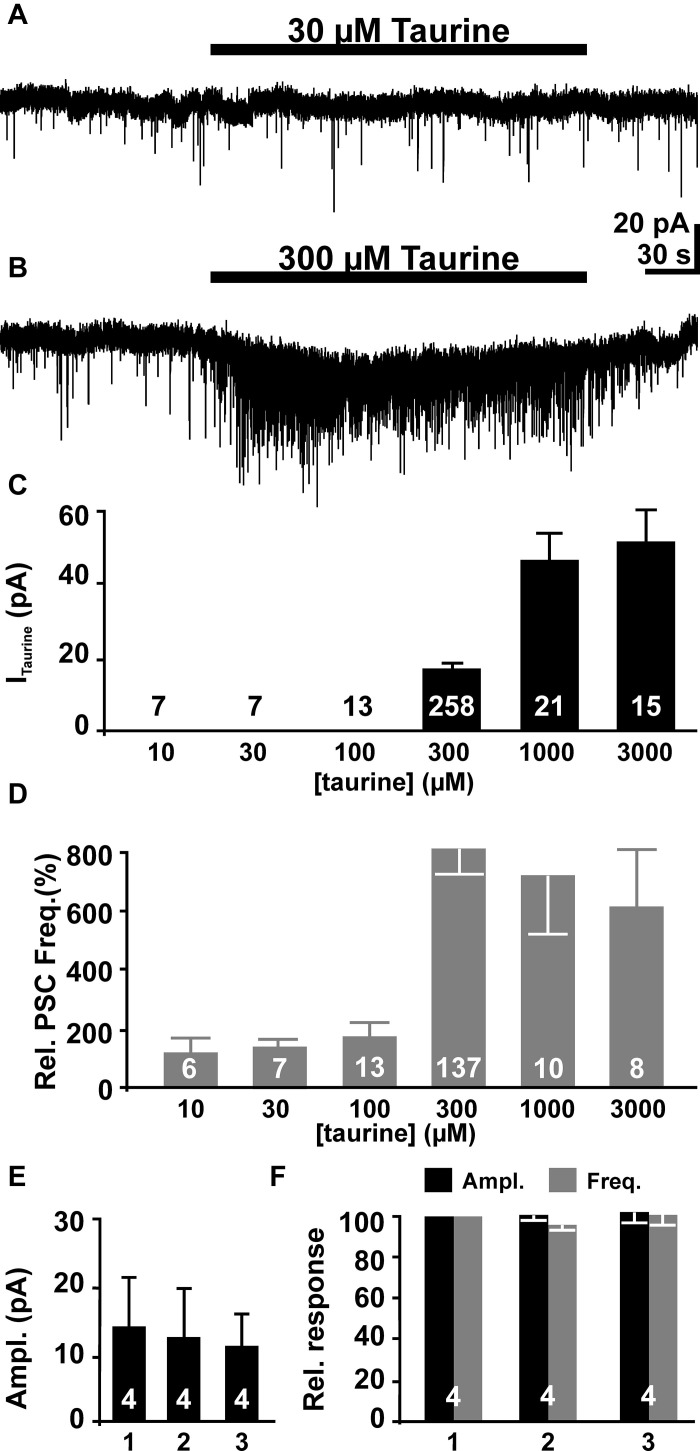
**The effect of bath-applied taurine on pyramidal neurons**. **(A)** Whole-cell voltage-clamp recording from a pyramidal neuron. Bath application of 30 μM taurine did not affect the holding current or the frequency of the PSCs. **(B)** Bath application of 300 μM taurine induced an inward current and a massive increase in the frequency of PSCs. **(C)** Statistical analysis of inward current amplitudes induced by different taurine concentration. **(D)** Statistical analysis of relative PSC frequency (normalized to control intervals) in the presence of different taurine concentrations. Note that the effect of taurine on PSC frequency displays a clear threshold behavior. **(E, F)** Neither the amplitude of the inward current **(E)**, nor amplitude (black bars) and frequency (gray bars) of taurine-induced PSCs differ significantly in three consecutive applications. Bars represent mean ± SEM, number of experiments is given in the bars.

Bath application of taurine induced an inward current in pyramidal neurons. While taurine concentration ≤ 100 μM did not elicit significant membrane currents (Figures 3A, C), inward currents of 17.3 ± 0.8 pA (*n* = 258), 46.9 ± 7.4 (*n* = 20) and 52 ± 8.8 pA (*n* = 15) were induced by bath application of 300 μM, 1 mM and 3 mM taurine, respectively (Figures 3B, C). The taurine-induced current showed a pronounced desensitization by 27.5 ± 3.8% and 31.4 ± 9% in 1 mM and 3 mM taurine, respectively, while no obvious desensitization occurred at lower taurine concentrations.

In addition to this inward current, bath application of taurine increased the frequency of PSCs in a considerable fraction of pyramidal neurons. While no significant effect was observed at taurine concentration ≤ 100 μM (Figures 3A, C), in 46.8% (133 out of 275 cells) of all investigated neurons bath application of 300 μM taurine induced an increase in the frequency of PSCs (Figure 3B). In 125 of these cells a significant (>4x SD of control) increase of PSC frequency by 815 ± 86% from 0.16 ± 0.02 Hz (*n* = 138) to 0.59 ± 0.05 Hz (*n* = 125, Figure 3D) was induced. In the eight additional neurons that showed PSCs during taurine application, PSCs were absent during the control interval. The majority (91.5%) of the remaining 142 neurons did no show PSCs neither before nor in the presence of 300 μM taurine, while in 12 neurons no significant increase in PSC frequency (between 89 and 229% of control) was observed in the presence of 300 μM taurine. The frequency of taurine-induced PSCs showed no significant correlation (*r*^2^ = 0.17) with the PSC frequency during the control interval. In addition, the amplitude of PSCs increased from 19.3 ± 1 pA (*n* = 137) to 24.6 ± 1.3 pA (*n* = 145) in the presence of 300 μM taurine. Furthermore, increasing the taurine concentration to 1 mM or 3 mM induced comparable effects on PSCs. Bath application of 1 mM taurine increased the frequency of PSCs in 10 out of 21 investigated neurons by 694 ± 210%, while 3 mM taurine increased the frequency in 6 out of 15 neurons and 518 ± 198% (*n* = 7, Figure 3D). Neither the amplitude nor the frequency was significantly different from the effects of 300 μM taurine, indicating that 300 μM taurine was sufficient to provoke a maximal effect on PSCs. Therefore we performed all subsequent experiments with 300 μM taurine. To test if taurine responses are stable during repetitive applications we applied 300 μM taurine for three times to the same cell. These experiments revealed no significant differences in taurine-induced inward currents (Figure 3E) as well as in amplitude and frequency of taurine-induced PSCs between the first, second and third taurine application (Figure 3F). In summary, these results show that bath application of taurine at a concentration ≥ 300 μM increased the frequency of the PSCs in about half of all pyramidal neurons, suggesting that activity of GABAergic synapses was considerably increased by taurine.

### Pharmacology of taurine-induced responses in pyramidal neurons

**Figure 4:**
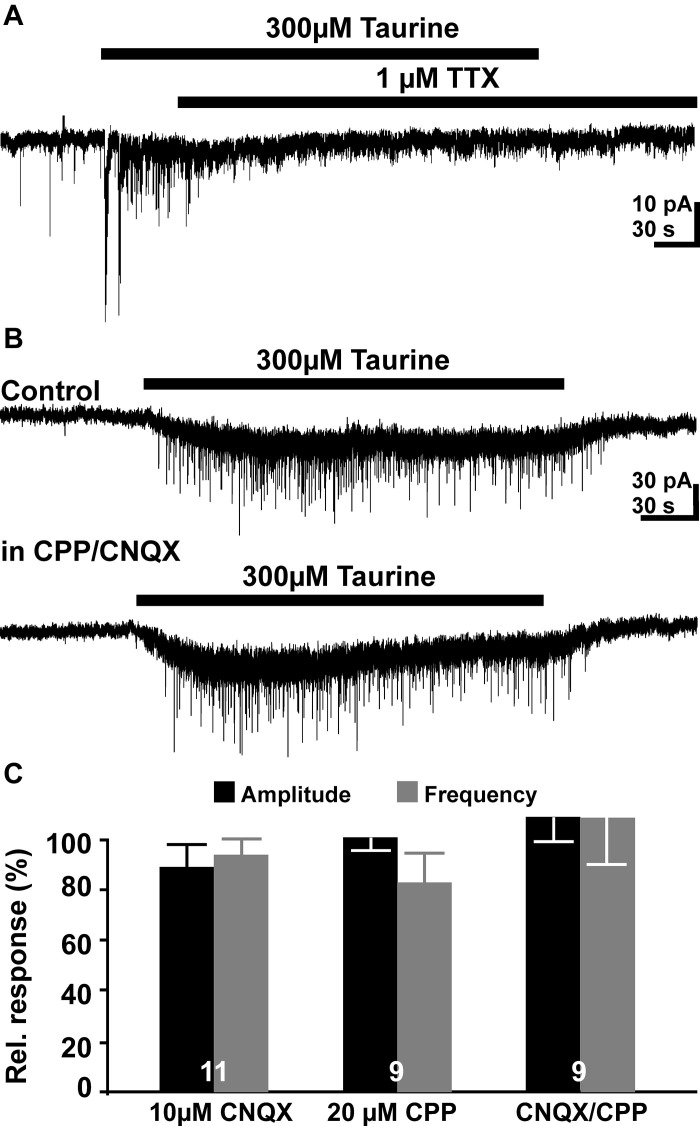
**Pharmacology of taurine responses**. **(A)** Typical current registration illustrating that taurine-induced increase in the PSC frequency was suppressed in the presence of 1 μM TTX. **(B)** Typical current registration illustrating that the taurine-induced increase in PSCs frequency was not affected in the presence of 10 μM CNQX 6-cyano-7- nitroquinoxaline-2,3-dione (CNQX) + 20 μM (±) R(-)-3-(2-carboxypiperazine-4-yl)-propyl-1-phosphonic acid (CPP). **(C)** Statistical analysis of the effect of glutamatergic antagonists on relative frequency and amplitude of taurine-induced PSCs. Bars represent mean ± SEM, numbers of experiments are given in the bars.

Next we analyzed the pharmacological properties of the PSCs evoked by the bath application of 300 μM taurine. Bath-application of TTX inhibited the taurine-induced PSCs (Figure 4A). In the presence of 0.2–1 μM TTX bath application of 300 μM taurine fails to increase the frequency (101.6 ± 14.3%, *n* = 6) and amplitude (97.4 ± 5%) of PSCs. In three additional TTX-treated slices that did not show spontaneous PSCs under control conditions, 300 μM taurine failed to evoke PSCs. The taurine-induced inward current was, however, not significantly affected under this condition (137 ± 25.1%, *n* = 9). These observations suggest that the taurine-induced increase in PSC frequency requires the activation of presynaptic networks.

Inhibition of ionotropic glutamate receptors by bath application of 10 μM CNQX or 20 μM CPP had no significant effect on the taurine-induced PSCs. In the presence of both glutamate antagonists neither amplitude (27.13 ± 3 vs. 31.6 ± 6.3 pA) nor the frequency (0.55 ± 0.09 vs. 0.55 ± 0.11 Hz) of taurine-induced PSCs was significantly (*p* = 0.5 and *p* = 1, respectively, *n* = 9) different from the control application in ACSF (Figures 4B, C). The taurine-induced inward current was also unaffected under this condition (15.9 ± 2.1 vs. 17.7 ± 4.3 pA, *n* = 9). Similar results were obtained if these glutamate receptor antagonists were applied alone (Figure 4C). These results suggest that glutamatergic synapses are not activated by bath application of taurine. These experiments also revealed that the frequency of spontaneous postsynaptic events (sPSCs) was not significantly reduced in the presence of 10 μM CNQX and 20 μM CPP (128 ± 40%, *n* = 9).

**Figure 5:**
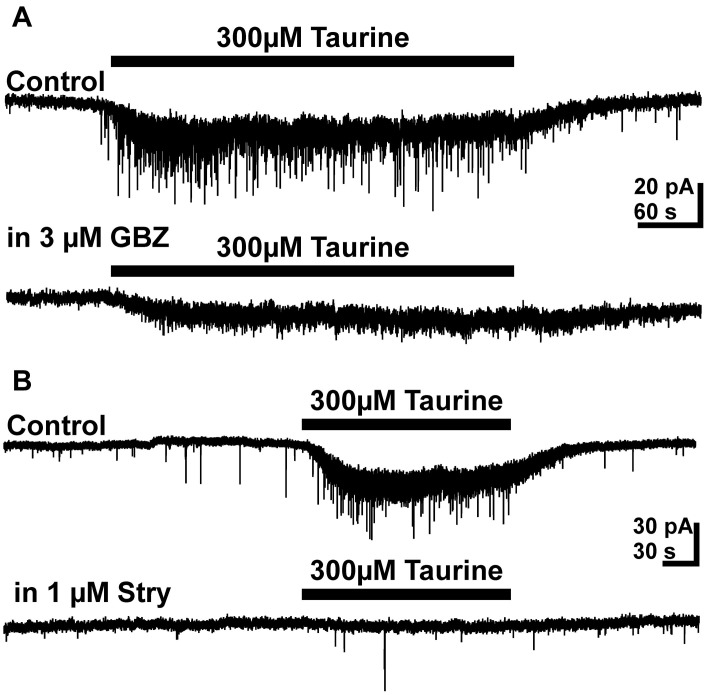
**Effect of GABA_A_ and glycine receptor antagonists on taurine induced responses. (A)** Typical current traces illustrating that 3 μM gabazine (GBZ) abolished the taurine-induced increase in PSC frequency and reduced the amplitude of the taurine-induced inward current. **(B)** Typical current traces illustrating that 1 μM strychnine abolished the taurine-induced increase in PSC frequency and reduced the amplitude of the taurine-induced inward current.

On the other hand, bath application of the GABA_A_ receptor antagonist gabazine (3 μM) completely blocked sPSCs in 19 out of 26 pyramidal cells investigated and massively reduced the frequency of sPSCs to 0.023 ± 0.007 Hz in the remaining seven neurons. This result indicates that the majority of sPSCs is mediated by GABA_A_ receptors. Bath application of 300 μM taurine in the presence of 3 μM gabazine did not induce a significant (*p* = 0.34) increase in frequency of PSCs (0.06 ± 0.02 Hz, *n* = 5, Figure 5A), suggesting that GABA_A_ receptors are required for this taurine effect. The taurine-induced inward current was significantly (*p* < 0.001) reduced by 79.6 ± 8.3% (*n* = 8) from 21 ± 2.6 to 5.6 ± 2.5 pA in the presence of gabazine.

Bath application of the glycine receptor antagonist strychnine (0.3–1 μM) had no significant effect on frequency (107 ± 15%, *n* = 12) and amplitude (92 ± 11%) of sPSCs, supporting previous reports that glycine receptors are not directly involved in synaptic transmission in the developing neocortex (Flint et al., 1998; Okabe et al., 2004; Kilb et al., 2008). However, in the presence of 0.3–1 μM strychnine the taurine-induced increase in PSC frequency was completely suppressed (Figure 5B). Under this condition bath application of taurine had no effect on the frequency (0.11 ± 0.01 vs. 0.11 ± 0.02 Hz, *n* = 12) and amplitude (13.84 ± 0.9 vs. 16.3 ± 2.4 pA, *n* = 12) of PSCs. In addition, the taurine-induced inward current was significantly (*p* < 0.001) reduced by 47.7 ± 8.3% (*n* = 12) from 22.2 ± 2.8 to 9.7 ± 1.1 pA. In the presence of both, 1 μM strychnine and 3 μM gabazine the taurine-induced inward current was massively reduced by 95.5 ± 1.2% to 1.1 ± 0.4 pA (*n* = 12). Under this condition bath application of 300 μM taurine did also not increase the frequency of PSCs (0.03 ± 0.004 vs. 0.03 ± 0.002, *n* = 8). In summary, these results suggest that glycine receptors are essential for the presynaptic taurine effect and also contribute to the postsynaptic taurinergic currents.

**Figure 6:**
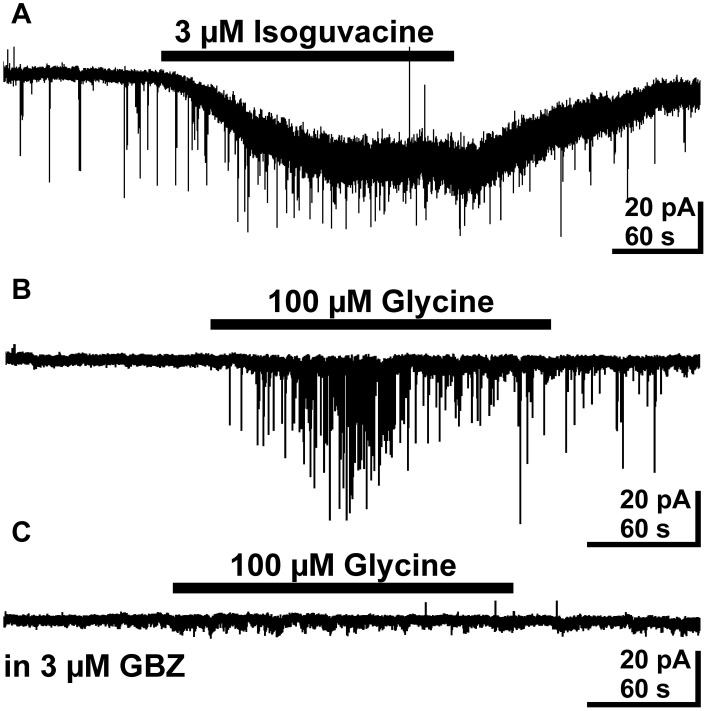
**Effect of bath applied isoguvacine and glycine on pyramidal neurons in the presence of 10 μM CNQX and 20 μM CPP**. **(A)** Typical current recording demonstrating that bath application of 3 μM isoguvacine induced an inward current and increased the PSC frequency. **(B)** Typical trace illustrating that bath application of 100 μM glycine increased the frequency of PSCs. **(C)** In the subsequent recording from the same neuron as in **B** the PSCs induced by bath application of 100 μM glycine are completely suppressed in the presence of 3 μM gabazine (GBZ).

Since the previous results indicate that taurine acting tonically on presynaptic glycine receptors enhance the frequency of putative GABAergic PSCs, we next directly stimulated presynaptic GABA_A_ and glycine receptors. These experiments were performed in the presence of 10 μM CNQX and 20 μM CPP to isolate GABAergic PSCs. Tonic activation of GABA_A_ receptors by bath application of 3 μM isoguvacine induced an inward current of 19.8 ± 5.3 pA (*n* = 8). In addition, it increased the frequency of PSCs by 566 ± 219% from 0.1 ± 0.04 to 0.24 ± 0.05 Hz (*n* = 8, Figure 6A). This result indicates that tonic activation of presynaptic GABA_A_ receptors is sufficient to enhance GABAergic inputs to pyramidal cells. Bath application of 100 μM glycine induced an inward current of 14.1 ± 4.8 pA (*n* = 6) and increased the frequency of PSCs in 4 out of 6 investigated pyramidal neurons by 1045 ± 831% (*n* = 4, Figure 6B) and induced in the remaining two neurons, which did not showed more than two PSCs in the control interval, a burst of PSCs. The frequency of glycine-induced PSCs was 0.47 ± 0.19 Hz (*n* = 6). To prove that these glycine-induced PSCs were indeed mediated by GABA_A_ receptors in the postsynaptic membrane, we next applied 100 μM glycine in the continuous presence of 3 μM gabazine, 10 μM CNQX and 20 μM CPP. Under this condition spontaneous PSCs were absent and bath application of 100 μM glycine did not induce PSCs (*n* = 6, Figure 6C), suggesting that glycine application enhanced exclusively GABAergic PSCs. In summary, these results indicate that activation of presynaptic glycine but also GABA_A_ receptors can increase the frequency of GABAergic PSCs.

### Taurine induced GABAergic postsynaptic currents (PSCs) that mediate excitatory responses

**Figure 7:**
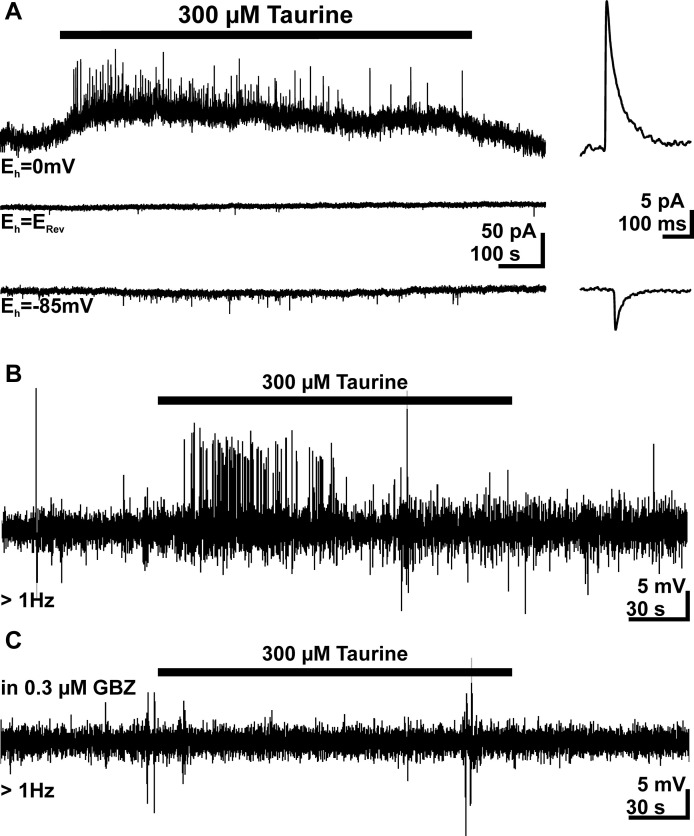
**Taurine induces Cl^-^-dependent, excitatory PSCs. (A)** Current traces recorded at a holding potential (*E*_h_) of 0 mV (upper trace) and −85 mV (bottom trace). For the middle trace *E*_h_ was set exactly at the GABA reversal potential (*E*_Rev_), which was experimentally determined by focal GABA pulses. There experiments were performed with a low Cl^-^ concentration in the pipette. Note that no taurine-induced increase in PSC frequency is visible if *E*_h_ = *E*_Rev_ and that the PSCs reverse their direction at this potential. Averaged PSCs are displayed at higher temporal resolution in the right panel. **(B)** Typical trace of a cell-attached recording from a pyramidal neuron. Bath application of 300 μM taurine induced APs. **(C)** In the subsequent cell-attached recording from the same neuron as in **B** the taurine-induced APs are completely suppressed in the presence of 0.3 μM gabazine (GBZ).

To strengthen our previous suggestion, that GABA_A_ receptors are underlying the taurine-induced PSCs in pyramidal neurons, we next determined the reversal potential (*E*_Rev_) of these PSCs. For this purpose we performed voltage-clamp experiments using a pipette-solution with a Cl^-^ concentration of 8 mM, which corresponds to an *E*_Rev_ of −62.5 mV. In these experiments the cell was clamped exactly to *E*_Rev_, which was determined experimentally for each cell by a series of focal GABA pulses at different holding potentials. These experiments revealed that the frequency of sPSCs was reduced to from 0.042 ± 0.013 Hz (*n* = 7) to 0.021 ± 0.006 Hz (*n* = 7) if the membrane was clamped from −85 mV to *E*_Rev_, indicating that a substantial fraction of sPSCs is mediated by ligand-gated Cl^-^ channels. At this holding potential bath application of 300 μM taurine had no significant (*p* = 0.35) effect on the frequency of PSCs (77 ± 15%, *n* = 5, Figure 7A). In contrast, under conditions when the holding potential was negative (−85 mV) or positive (0 mV) to *E*_Rev_ bath application of 300 μM taurine significantly increased the frequency of PSCs to 454 ± 70% (*n* = 7) and 630 ± 149% (*n* = 11), respectively, indicating that taurine initiates PSCs that are mediated via ligand-gated Cl^-^ channels. Accordingly the taurine-induced PSCs reversed at −56.7 mV, approximately at the estimated *E*_Rev_ (Figure 7B).

As many studies indicate that an activation of GABA_A_ receptors mediate depolarizing membrane responses in immature neocortical neurons (Owens et al., 1996; Achilles et al., 2007; Kolbaev et al., 2011b), we next investigated whether taurine-induced membrane responses mediate excitatory actions under physiological conditions. Therefore we performed cell-attached recordings to investigate this effect under conditions in which the intracellular Cl^-^ concentration ([Cl^-^]_*i*_) remained undisturbed. In 28 out of 35 investigated neurons spontaneous APs at a frequency of 0.29 ± 0.9 Hz (*n* = 28) were observed, the remaining eight cells showed less that three APs during the control interval. Bath application of 300 μM taurine increased the AP frequency in 20 (corresponding to 57%) of these neurons by more than 50% to 0.68 ± 0.16 Hz (Figure 7B). In 15 of the cells the AP frequency was sufficiently high (>0.15 Hz, corresponding to >25 spikes per control interval) to estimate possible inhibitory action. However, in only 3 of these 15 cells bath-application of taurine reduced AP frequency. In order to unravel, whether the taurine-induced increase in AP frequency was caused by GABAergic PSCs or the depolarizing inward current, we next applied 300 μM taurine in the presence of low gabazine concentrations, which selectively inhibit phasic GABAergic currents (Stell and Mody, 2002; Kolbaev et al., 2012). Accordingly we were able to show that bath application of 0.3 μM gabazine completely blocked the taurine-induced increase in the frequency of GABAergic PSCs (*n* = 7), while the tonic current was not significantly affected (94.9 ± 16.3%, *n* = 7). Cell-attached recordings revealed that in the presence of 0.3 μM gabazine bath application of 300 μM taurine had no effect on AP frequency in 12 investigated cells, which all revealed a taurine-induced increase in AP frequency under control conditions (Figure 7C). These results strongly suggest that the taurine-induced PSCs are GABAergic and mediate an excitatory action on pyramidal neurons.

### Effect of taurine on GABAergic interneurons

**Figure 8:**
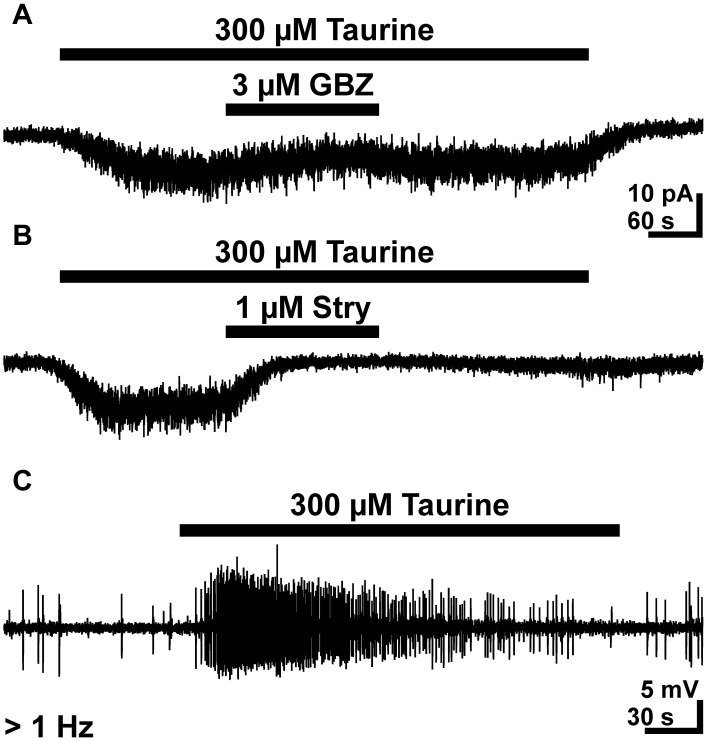
**Effect of taurine on GABAergic interneurons**. **(A)** Typical current registration illustrating that bath application of 300 μM taurine induced an inward current that was attenuated by 3 μM gabazine (GBZ). **(B)** The taurine-induced inward currents revealed a stronger attenuation in the presence of 1 μM strychnine. **A** and **B** were recorded in the continuous presence of 0.5 μM TTX. **(C)** Typical cell-attached recording illustrating that bath-application of 300 μM taurine increases the frequency of APs in GABAergic interneurons.

Since the previous results demonstrated that taurine enhanced the frequency of GABAergic PSCs, we next analyzed the effect of taurine on GABAergic interneurons. Therefore we performed whole-cell and cell-attached measurements on GABAergic interneurons identified by GFP expression under the control of GAD67 (Tamamaki et al., 2003). The whole-cell experiments revealed that bath application of 300 μM taurine induced an inward current of 10.6 ± 2.2 pA (*n* = 14). This inward current was significantly attenuated by 49 ± 8% (*n* = 6, *p* = 0.0313) in the presence of 3 μM gabazine and by 70 ± 13% (*n* = 7, *p* = 0.0156) in the presence of 1 μM strychnine (Figures 8A, B). In summary, these results indicate that taurine mediates a tonic activation of glycine receptors and to a lesser extent of GABA_A_ receptors in these cells.

To analyze whether this taurine-induced inward current mediates an excitatory effect under conditions with undisturbed [Cl^-^]_i_, we next performed cell-attached experiments. These experiments revealed that bath application of 300 μM taurine induced in 17 out of 30 investigated GABAergic interneurons (corresponding to 56.7%) a significant (*p* < 0.001) increase in AP frequency from 0.1 ± 0.02 to 1.1 ± 0.27 Hz (Figure 8C). In summary, these results demonstrate that bath application of 300 μM taurine excites a considerably fraction of GABAergic interneurons and that this activity is mediated mainly via glycine receptors.

**Figure 9:**
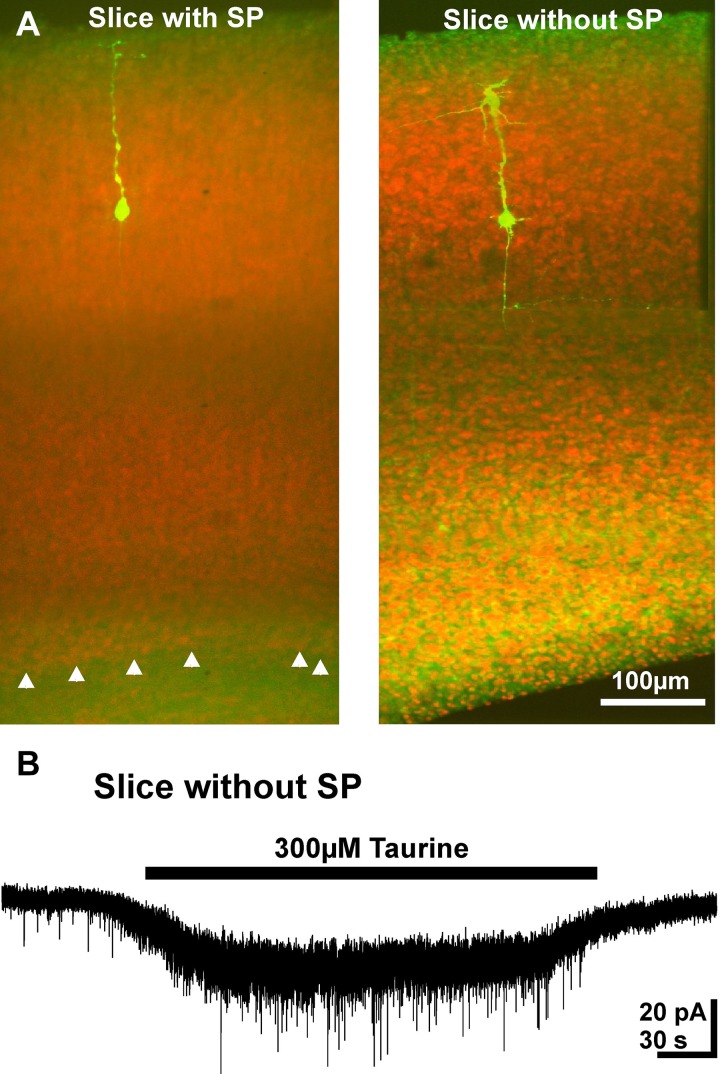
**Taurine effect on pyramidal neurons in reduced slice preparation**. **(A)** Confocal images of biocytin-labeled neurons (green) counter-stained with propidium jodide. Note the absence of the SP (white arrowheads) in the right slice. **(B)** Typical current registration illustrating that bath application of taurine increased the frequency of PSCs in a reduced slice preparation without SP.

Finally, we investigated whether SP cells, which are comprised of a significant fraction of GABAergic neurons (Kanold and Luhmann, 2010) and are activated by taurine (Kilb et al., 2008), contribute to GABAergic taurine-induced PSCs. For this purpose we used a reduced neocortical slice preparations either containing or lacking the SP (Figure 9A). In both preparations bath application of 300 μM taurine induced PSC activity. In reduced slices that contained the SP, taurine increased the frequency of PSCs by 531 ± 138% (*n* = 4) to 0.19 ± 0.02 Hz. A comparable effect was observed in slices without SP, in which the frequency of PSCs increased by 697 ± 170% (*n* = 6) to 0.26 ± 0.05 Hz (Figure 9B). The taurine-induced PSCs had comparable amplitudes in both preparations (14.4 ± 1.9 vs. 18.8 ± 1.8 pA). In summary, these results suggest that GABAergic SP neurons do not contribute substantially to the taurine-induced activity.

## Discussion

The main findings of this study can be summarized as follows: (i) bath application of 300 μM taurine increased the frequency of PSCs in a considerable fraction of pyramidal neurons; (ii) taurine, but also glycine and isoguvacine specifically increased the frequency of GABA_A_ receptor mediated PSCs; (iii) these taurine-induced GABAergic PSCs mediated an excitatory effect; (iv) taurine, acting mainly on glycine receptors, induced an inward current and repetitive APs in GABAergic interneurons; and (v) GABAergic SP neurons do not substantially contribute to the taurine-induced activity. We conclude from these results that taurine can enhance the activity of excitatory GABAergic networks in the immature neocortex.

Passive and active membrane properties of the pyramidal neurons from P2–P4 mice are in accordance with previous results (Kriegstein et al., 1987; Luhmann et al., 2000; Picken Bahrey and Moody, 2003; Okabe et al., 2004). The passive membrane properties of the immature GABAergic interneuron as well as the relatively low maximal firing frequency of these neurons are in line with the developmental profile of immature interneurons (Doischer et al., 2008; Okaty et al., 2009).

One major conclusion of our study is that taurine specifically increased the frequency of GABAergic PSCs. Several lines of evidence support this hypothesis. First, the taurine-mediated increase in PSC frequency was unaffected by glutamatergic antagonists but completely suppressed by the GABA_A_ receptor antagonist gabazine. This finding may be explained by the hypothesis that GABA_A_ receptor activation on presynaptic cells is required for the activation of the PSCs, but the observation that bath-application of glycine induced a similar gabazine-sensitive increase in PSC frequency suggests that a taurine-mediated glycine receptor activation is sufficient to induce these PSCs. Second, the taurine-induced PSCs reversed around *E*_Cl_, which would, however, also match to a glycinergic neurotransmission. But the observation that the glycine-induced increase in PSC frequency was completely blocked by gabazine indicates that under this condition only GABAergic PSCs were induced and suggests that this is also the case for the taurine-induced PSCs. These suggestions are supported by the observation that an adequate glycinergic stimulation of presynaptic cells is required to provoke taurine-induced PSCs. And finally, the observation that a specific inhibition of synpatically GABA_A_ receptors by low gabazine concentrations completely abolished the taurine-induced PSCs also indicates that these PSCs were GABAergic. In summary, these results strongly suggest that bath-application of taurine, but also of glycine or isoguvacine, selectively activated GABAergic PSCs.

A variety of previous studies demonstrated that activation of GABA_A_ receptors mediate depolarizing membrane responses in immature neurons, due to the high [Cl^-^]_*i*_ in these cells (Owens et al., 1996; Achilles et al., 2007; Ben-Ari et al., 2012). Although such depolarizing responses do not necessarily enhance the excitability of neurons (Jedlicka et al., 2011; Kolbaev et al., 2011a), our observation that under cell-attached conditions taurine can induce barrages of APs, which are abolished by a specific inhibition of synaptic GABA­ergic currents with a low dose of gabazine, convincingly demonstrate that these synaptic GABA­ergic inputs mediate an excitation of immature pyramidal neurons. This is in line with previous publications which reported that in the immature neocortex GABA­ergic inputs from the zona incerta can mediate excitatory responses in pyramidal neurons (Dammerman et al., 2000) and that in immature GABAergic interneurons and Cajal-Retzius cells GABAergic synaptic inputs reliably trigger APs (Rheims et al., 2008; Cosgrove and Maccaferri, 2012).

Theoretically, the GABAergic PSCs can rely on an increased spontaneous release probability of GABA vesicles or from an excitation of presynaptic GABAergic interneurons (Hessler et al., 1993; Kirmse and Kirischuk, 2006). However, as the taurine-induced increase in PSCs frequency was absent in the presence of TTX, these PSCs reflect an increased activity of GABAergic interneurons rather than an increased release probability of presynaptic neurotransmitter vesicles. Accordingly, we were able to directly demonstrate that taurine induced an inward current in GABAergic interneurons, which is in 56% of these neurons sufficient to trigger APs. Interestingly the frequency of the APs is higher than the frequency of the taurine-induced PSCs in pyramidal neurons, suggesting a high failure rate and/or synaptic fatigue in these GABAergic synapses, but probably also that only few GABAergic interneurons make synaptic contacts to pyramidal neurons at this age. The taurinergic inward current in GABAergic interneurons was mimicked by bath application of glycine and isoguvacine, indicating that both GABA_A_ and glycine receptors can potentially contribute to the taurine effect. The puzzling observation that in interneurons strychnine abolished responses to focal taurine application, while the tonic current mediated by bath applied taurine was only reduced by 70%, may be caused by distinct activation kinetics of glycine and GABA receptors upon fast application and by a differential desensitization of both receptors in the continuous presence of taurine. While GABA_A_ receptors had already been described in immature interneurons (Gozlan and Ben-Ari, 2003), no evidences for glycine receptors on cortical interneurons were reported so far. The observations that strychnine completely blocked focal taurine responses and suppressed bath-applied responses by 70%, while gabazine had a lower efficiency, suggest that taurine exerts its effect on GABAergic interneurons mainly via glycine receptors. Accordingly, the taurine-induced GABAergic PSCs were abolished in the presence of strychnine, illustrating that a taurinergic activation of only GABA_A_ receptors was insufficient to activate the presynaptic GABAergic networks.

The subtype of the GABAergic interneurons that mediate these excitatory synaptic inputs could not be defined in our study. We can exclude that a GABAergic population of SP neurons (Hanganu et al., 2002; Luhmann et al., 2009; Kanold and Luhmann, 2010) is a major source of the taurine-induced GABAergic PSCs, since we could demonstrate that even in a reduced slice preparation, which lacks a SP, taurine increases the frequency of PSCs. Although different subpopulations of inhibitory interneurons can be identified in the mature neocortex based on molecular, morphological and electrophysiological parameters (Markram et al., 2004; Ascoli et al., 2008), the late development of these markers (Doischer et al., 2008; Okaty et al., 2009) does not allow a reliably identification of the prospective subtype of the recorded interneurons during early postnatal development. The intriguing question, whether specific GABAergic interneurons transiently integrate into immature excitatory circuits thus remains elusive. Interestingly, GABAergic interneurons receive reliably excitatory GABAergic inputs (Rheims et al., 2008), while at least in the somatosensory neocortex no reliably excitatory inputs from the thalamus were found in early postnatal stages (Daw et al., 2007), suggesting that GABAergic interneurons are integrated into transient, mainly GABAergic circuits that mediate excitation rather than the feed-forward inhibition typical for mature neocortical circuits. One intriguing observation of the present study is that a taurine-induced increase in PSC frequency was observed only in 46% of the investigated pyramidal neurons, indicating that only a portion of the pyramidal neurons was integrated into such functional GABAergic networks.

In the immature hippocampus early generated pioneer GABAergic interneurons synchronize network activity transients (Bonifazi et al., 2009; Picardo et al., 2011). Such spontaneous early activity transients occur in most developing neuronal networks and are supposed to promote the formation of functional networks (Spitzer, 2006; Hanganu-Opatz, 2010; Kilb et al., 2011). In the neocortex, however, spontaneous network activity or oscillatory events are mostly independent of GABA_A_ receptors (Garaschuk et al., 2000; Dupont et al., 2006; Allene et al., 2008), although in ventral aspects of the neocortex a contribution of GABA_A_ receptors to spontaneous activity has been reported in the first postnatal week (Conhaim et al., 2011). Therefore we propose that this GABAergic excitatory network does not directly contribute to large-scale activity transients, but controls the excitability of specific, probably more local excitatory networks.

Finally, it has to be considered that the taurine concentration of 300 μM used in the present study most likely exceeds the interstitial taurine levels that can be expected in the immature neocortex. However, in rat and human fetal brains taurine is the most abundant neurotransmitter (Das and Ray, 1997; Benitez-Diaz et al., 2003). It has been suggested that taurine can be released from immature neurons after electrical stimulation or by hypoosmolar solution (Flint et al., 1998; Kilb et al., 2008) by non-synaptic processes, e.g., by volume-sensitive organic osmolyte channels or reversal of taurine transport (Ando et al., 2012). Therefore we propose that such an activity-dependent non-synaptic release of taurine can led to subtle changes in the interstitial taurine concentrations, which in turn slightly increase the excitability of immature networks and may thereby directly influence the maturation of neocortical neuronal circuits.

## Author contributions

Werner Kilb designed the experiments, Bogdan A. Sava, Rongqing Chen and Haiyan Sun performed the experiments, Bogdan A. Sava and Werner Kilb analyzed the data, Heiko J. Luhmann and Werner Kilb wrote the manuscript.

## Conflict of interest statement

The authors declare that the research was conducted in the absence of any commercial or financial relationships that could be construed as a potential conflict of interest.

## References

[B1] Achilles K. Okabe A. Ikeda M. Shimizu-Okabe C. Yamada J. Fukuda A. (2007). Kinetic properties of Cl uptake mediated by Na^+^-dependent K^+^-2Cl cotransport in immature rat neocortical neurons. J. Neurosci. 27, 8616–8627 10.1523/jneurosci.5041-06.2007 17687039 PMC6672936

[B2] Aguayo L. G. van Zundert B. Tapia J. C. Carrasco M. A. Alvarez F. J. (2004). Changes on the properties of glycine receptors during neuronal development. Brain Res. Brain Res Rev. 47, 33–45 10.1016/j.brainresrev.2004.06.007 15572161

[B3] Albrecht J. Schousboe A. (2005). Taurine interaction with neurotransmitter receptors in the CNS: an update. Neurochem. Res. 30, 1615–1621 10.1007/s11064-005-8986-6 16362781

[B4] Allene C. Cattani A. Ackman J. B. Bonifazi P. Aniksztejn L. Ben-Ari Y. (2008). Sequential generation of two distinct synapse-driven network patterns in developing neocortex. J. Neurosci. 28, 12851–12863 10.1523/jneurosci.3733-08.2008 19036979 PMC6671804

[B5] Ando D. Kubo Y. Akanuma S. Yoneyama D. Tachikawa M. Hosoya K. (2012). Function and regulation of taurine transport in Muller cells under osmotic stress. Neurochem. Int. 60, 597–604 10.1016/j.neuint.2012.02.018 22391325

[B6] Ascoli G. A. Alonso-Nanclares L. Anderson S. A. Barrionuevo G. Benavides-Piccione R. Burkhalter A. (2008). Petilla terminology: nomenclature of features of GABAergic interneurons of the cerebral cortex. Nat. Rev. Neurosci. 9, 557–568 10.1038/nrn2402 18568015 PMC2868386

[B7] Behar T. N. Schaffner A. E. Scott C. A. Greene C. L. Barker J. L. (2000). GABA receptor antagonists modulate postmitotic cell migration in slice cultures of embryonic rat cortex. Cereb. Cortex 10, 899–909 10.1093/cercor/10.9.899 10982750

[B8] Behar T. N. Smith S. V. Kennedy R. T. McKenzie J. M. Maric I. Barker J. L. (2001). GABA(B) receptors mediate motility signals for migrating embryonic cortical cells. Cereb. Cortex 11, 744–753 10.1093/cercor/11.8.744 11459764

[B9] Ben-Ari Y. (2002). Excitatory actions of GABA during development: the nature of the nurture. Nat. Rev. Neurosci. 3, 728–739 10.1038/nrn920 12209121

[B10] Ben-Ari Y. (2006). Basic developmental rules and their implications for epilepsy in the immature brain. Epileptic Disord. 8, 91–102 16793570

[B11] Ben-Ari Y. (2012). The yin and yen of GABA in brain development and operation in health and disease. Front. Cell. Neurosci. 6:45 10.3389/fncel.2012.00045 23162428 PMC3494101

[B12] Ben-Ari Y. Woodin M. A. Sernagor E. Cancedda L. Vinay L. Rivera C. (2012). Refuting the challenges of the developmental shift of polarity of GABA actions: GABA more exciting than ever! Front. Cell. Neurosci. 6:35 10.3389/fncel.2012.00035 22973192 PMC3428604

[B13] Ben-Ari Y. Cherubini E. Corradetti R. Gaiarsa J.-L. (1989). Giant synaptic potentials in immature rat CA3 hippocampal neurones. J. Physiol. 416, 303–325 2575165 10.1113/jphysiol.1989.sp017762 PMC1189216

[B14] Benitez-Diaz P. Miranda-Contreras L. Mendoza-Briceno R. V. Pena-Contreras Z. Palacios-Pru E. (2003). Prenatal and postnatal contents of amino acid neurotransmitters in mouse parietal cortex. Dev. Neurosci. 25, 366–374 10.1159/000073514 14614264

[B15] Bonifazi P. Goldin M. Picardo M. A. Jorquera I. Cattani A. Bianconi G. (2009). GABAergic hub neurons orchestrate synchrony in developing hippocampal networks. Science 326, 1419–1424 10.1126/science.1175509 19965761

[B16] Bormann J. Hamill O. P. Sakmann B. (1987). Mechanism of anion permeation through channels gated by glycine and gamma-aminobutyric acid in mouse cultured spinal neurones. J. Physiol. 385, 243–286 2443667 10.1113/jphysiol.1987.sp016493 PMC1192346

[B17] Carlson V. C. C. Yeh H. H. (2011). GABA(A) receptor subunit profiles of tangentially migrating neurons derived from the medial ganglionic eminence. Cereb. Cortex 21, 1792–1802 10.1093/cercor/bhq247 21148088 PMC3202737

[B18] Conhaim J. Easton C. R. Becker M. I. Barahimi M. Cedarbaum E. R. Moore J. G. (2011). Developmental changes in propagation patterns and transmitter dependence of waves of spontaneous activity in the mouse cerebral cortex. J. Physiol. 589, 2529–2541 10.1113/jphysiol.2010.202382 21486817 PMC3115823

[B19] Cosgrove K. E. Maccaferri G. (2012). mGlu1 alpha-dependent recruitment of excitatory GABAergic input to neocortical Cajal-Retzius cells. Neuropharmacol. 63, 486–493 10.1016/j.neuropharm.2012.04.025 22579657 PMC3372658

[B20] Cuzon V. C. Yeh P. W. Cheng Q. Yeh H. H. (2006). Ambient GABA promotes cortical entry of tangentially migrating cells derived from the medial ganglionic eminence. Cereb. Cortex 16, 1377–1388 10.1093/cercor/bhj084 16339085

[B21] Dammerman R. S. Flint A. C. Noctor S. Kriegstein A. R. (2000). An excitatory GABAergic plexus in developing neocortical layer 1. J. Neurophysiol. 84, 428–434 10899216 10.1152/jn.2000.84.1.428

[B22] Das S. K. Ray P. K. (1997). Ontogeny of neurotransmitter amino acids in human fetal brains. Biochem. Mol. Biol. Int. 42, 193–202 10.1080/15216549700202581 9192100

[B23] Daw M. I. Ashby M. C. Isaac J. T. (2007). Coordinated developmental recruitment of latent fast spiking interneurons in layer IV barrel cortex. Nat. Neurosci. 10, 453–461 10.1038/nn1866 17351636

[B24] Dodt H.-U Zieglgänsberger W. (1990). Visualizing unstained neurons in living brain slices by infrared DIC-videomicroscopy. Brain Res. 537, 333–336 10.1016/0006-8993(90)90380-t 2085783

[B25] Doischer D. Hosp J. A. Yanagawa Y. Obata K. Jonas P. Vida I. (2008). Postnatal differentiation of basket cells from slow to fast signaling devices. J. Neurosci. 28, 12956–12968 10.1523/jneurosci.2890-08.2008 19036989 PMC6671784

[B26] Dupont E. Hanganu I. L. Kilb W. Hirsch S. Luhmann H. J. (2006). Rapid developmental switch in the mechanisms driving early cortical columnar networks. Nature 439, 79–83 10.1038/nature04264 16327778

[B27] Flint A. C. Liu X. L. Kriegstein A. R. (1998). Nonsynaptic glycine receptor activation during early neocortical development. Neuron 20, 43–53 10.1016/s0896-6273(00)80433-x 9459441

[B28] Fritschy J. M. Paysan J. Enna A. Mohler H. (1994). Switch in the expression of rat GABA_A_-receptor subtypes during postnatal development: an immunohistochemical study. J. Neurosci. 14, 5302–5324 8083738 10.1523/JNEUROSCI.14-09-05302.1994 PMC6577100

[B29] Garaschuk O. Linn J. Eilers J. Konnerth A. (2000). Large-scale oscillatory calcium waves in the immature cortex. Nat. Neurosci. 3, 452–459 10.1038/74823 10769384

[B30] Gozlan H. Ben-Ari Y. (2003). Interneurons are the source and the targets of the first synapses formed in the rat developing hippocampal circuit. Cereb. Cortex 13, 684–692 10.1093/cercor/13.6.684 12764045

[B31] Hanganu I. L. Kilb W. Luhmann H. J. (2002). Functional synaptic projections onto subplate neurons in neonatal rat somatosensory cortex. J. Neurosci. 22, 7165–7176 12177212 10.1523/JNEUROSCI.22-16-07165.2002 PMC6757868

[B32] Hanganu-Opatz I. L. (2010). Between molecules and experience: role of early patterns of coordinated activity for the development of cortical maps and sensory abilities. Brain Res. Rev. 64, 160–176 10.1016/j.brainresrev.2010.03.005 20381527

[B33] Haydar T. F. Wang F. Schwartz M. L. Rakic P. (2000). Differential modulation of proliferation in the neocortical ventricular and subventricular zones. J. Neurosci. 20, 5764–5774 10908617 10.1523/JNEUROSCI.20-15-05764.2000 PMC3823557

[B34] Heck N. Kilb W. Reiprich P. Kubota H. Furukawa T. Fukuda A. (2007). GABA_A_ receptors regulate neocortical neuronal migration in vitro and in vivo. Cereb. Cortex 17, 138–148 10.1093/cercor/bhj135 16452638

[B35] Hessler N. A. Shirke A. M. Malinow R. (1993). The probability of transmitter release at a mammalian central synapse. Nature 366, 569–572 10.1038/366569a0 7902955

[B36] Inoue K. Furukawa T. Kumada T. Yamada J. Wang T. Y. Inoue R. (2012). Taurine inhibits K^+^-Cl^-^ cotransporter KCC2 to regulate embryonic Cl^-^ homeostasis via With-no-lysine (WNK) protein kinase signaling pathway. J. Biol. Chem. 287, 20839–20850 10.1074/jbc.m111.319418 22544747 PMC3375508

[B37] Ito S. Cherubini E. (1991). Strychnine-sensitive glycine responses of neonatal rat hippocampal neurones. J. Physiol. 440, 67–83 1804982 10.1113/jphysiol.1991.sp018696 PMC1180140

[B38] Jedlicka P. Deller T. Gutkin B. S. Backus K. H. (2011). Activity-dependent intracellular chloride accumulation and diffusion controls GABA(A) receptor-mediated synaptic transmission. Hippocampus 21, 885–898 10.1002/hipo.20804 20575006

[B39] Ji F. Y. Kanbara N. Obata K. (1999). GABA and histogenesis in fetal and neonatal mouse brain lacking both the isoforms of glutamic acid decarboxylase. Neurosci. Res. 33, 187–194 10.1016/s0168-0102(99)00011-5 10211762

[B40] Kanold P. O. Luhmann H. J. (2010). The subplate and early cortical circuits. Annu. Rev. Neurosci. 33, 23–48 10.1146/annurev-neuro-060909-153244 20201645

[B41] Kilb W. Luhmann H. J. (2001). Spontaneous GABAergic postsynaptic currents in Cajal-Retzius cells in neonatal rat cerebral cortex. Eur. J. Neurosci. 13, 1387–1390 10.1046/j.0953-816x.2001.01514.x 11298799

[B42] Kilb W. Hanganu I. L. Okabe A. Sava B. A. Shimizu-Okabe C. Fukuda A. (2008). Glycine receptors mediate excitation of subplate neurons in neonatal rat cerebral cortex. J. Neurophysiol. 100, 698–707 10.1152/jn.00657.2007 18562558

[B43] Kilb W. Ikeda M. Uchida K. Okabe A. Fukuda A. Luhmann H. J. (2002). Depolarizing glycine responses in Cajal-Retzius cells of neonatal rat cerebral cortex. Neuroscience 112, 299–307 10.1016/s0306-4522(02)00071-4 12044448

[B44] Kilb W. Kirischuk S. Luhmann H. J. (2011). Electrical activity patterns and the functional maturation of the neocortex. Eur. J. Neurosci. 34, 1677–1686 10.1111/j.1460-9568.2011.07878.x 22103424

[B45] Kilb W. Kirischuk S. Luhmann H. J. (2013). Role of tonic GABAergic currents during pre- and early postnatal rodent development. Front. Neural Circ. 7:139 10.3389/fncir.2013.00139 24027498 PMC3760143

[B46] Kirmse K. Kirischuk S. (2006). Ambient GABA constrains the strength of GABAergic synapses at Cajal-Retzius cells in the developing visual cortex. J. Neurosci. 26, 4216–4227 10.1523/jneurosci.0589-06.2006 16624942 PMC6674013

[B47] Kolbaev S. N. Achilles K. Luhmann H. J. Kilb W. (2011a). Effect of depolarizing GABA(A)-mediated membrane responses on excitability of Cajal-Retzius cells in the immature rat neocortex. J. Neurophysiol. 106, 2034–2044 10.1152/jn.00699.2010 21775719

[B48] Kolbaev S. N. Luhmann H. J. Kilb W. (2011b). Activity-dependent scaling of GABA-ergic excitation by dynamic Cl(-) changes in Cajal-Retzius cells. Pflugers Arch. 461, 557–565 10.1007/s00424-011-0935-4 21336585

[B49] Kolbaev S. N. Sharopov S. Dierkes P. W. Luhmann H. J. Kilb W. (2012). Phasic GABAA-receptor activation is required to suppress epileptiform activity in the CA3 region of the immature rat hippocampus. Epilepsia 53, 888–896 10.1111/j.1528-1167.2012.03442.x 22432890

[B50] Kriegstein A. R. Suppes T. Prince D. A. (1987). Cellular and synaptic physiology and epileptogenesis of developing rat neocortical neurons in vitro. Brain Res. 431, 161–171 10.1016/0165-3806(87)90206-9 3040188

[B51] Laurie D. J. Wisden W. Seeburg P. H. (1992). The distribution of thirteen GABA_A_ receptor subunit mRNAs in the rat brain. III. Embryonic and postnatal development. J. Neurosci. 12, 4151–4172 1331359 10.1523/JNEUROSCI.12-11-04151.1992 PMC6576006

[B52] LoTurco J. J. Owens D. F. Heath M. J. Davis M. B. Kriegstein A. R. (1995). GABA and glutamate depolarize cortical progenitor cells and inhibit DNA synthesis. Neuron 15, 1287–1298 10.1016/0896-6273(95)90008-x 8845153

[B53] Luhmann H. J. Kilb W. Hanganu-Opatz I. L. (2009). Subplate cells: amplifiers of neuronal activity in the developing cerebral cortex. Front. Neuroanat. 3:19 10.3389/neuro.05.019.2009 19862346 PMC2766272

[B54] Luhmann H. J. Reiprich R. A. Hanganu I. Kilb W. (2000). Cellular physiology of the neonatal rat cerebral cortex: intrinsic membrane properties, sodium and calcium currents. J. Neurosci. Res. 62, 574–584 10.1002/1097-4547(20001115)62:4<574::aid-jnr12>3.0.co;2-0 11070501

[B55] Maar T. Moran J. Schousboe A. Pasantes-Morales H. (1995). Taurine deficiency in dissociated mouse cerebellar cultures affects neuronal migration. Int. J. Dev. Neurosci. 13, 491–502 10.1016/0736-5748(95)00068-r 7484220

[B56] Markram H. Toledo-Rodriguez M. Wang Y. Gupta A. Silberberg G. Wu C. (2004). Interneurons of the neocortical inhibitory system. Nat. Rev. Neurosci. 5, 793–807 10.1038/nrn1519 15378039

[B57] Oja S. S. Saransaari P. (2000). Modulation of taurine release by glutamate receptors and nitric oxide. Prog. Neurobiol. 62, 407–425 10.1016/s0301-0082(00)00005-8 10856611

[B58] Okabe A. Kilb W. Shimizu-Okabe C. Hanganu I. L. Fukuda A. Luhmann H. J. (2004). Homogenous glycine receptor expression in cortical plate neurons and Cajal-Retzius cells of neonatal rat cerebral cortex. Neuroscience 123, 715–724 10.1016/j.neuroscience.2003.10.014 14706783

[B59] Okaty B. W. Miller M. N. Sugino K. Hempel C. M. Nelson S. B. (2009). Transcriptional and electrophysiological maturation of neocortical fast-spiking GABAergic interneurons. J. Neurosci. 29, 7040–7052 10.1523/jneurosci.0105-09.2009 19474331 PMC2749660

[B60] Owens D. F. Boyce L. H. Davis M. B. Kriegstein A. R. (1996). Excitatory GABA responses in embryonic and neonatal cortical slices demonstrated by gramicidin perforated-patch recordings and calcium imaging. J. Neurosci. 16, 6414–6423 8815920 10.1523/JNEUROSCI.16-20-06414.1996 PMC6578913

[B61] Owens D. F. Liu X. L. Kriegstein A. R. (1999). Changing properties of GABA_A_ receptor-mediated signaling during early neocortical development. J. Neurophysiol. 82, 570–583 10444657 10.1152/jn.1999.82.2.570

[B62] Palackal T. Moretz R. Wisniewski H. Sturman J. (1986). Abnormal visual cortex development in the kitten associated with maternal dietary taurine deprivation. J. Neurosci. Res. 15, 223–239 10.1002/jnr.490150212 2421007

[B63] Paxinos G. Franklin K. B. J. (2001). The Mouse Brain in Stereotaxic Coordinates. San Diego: Academic Press

[B64] Picardo M. A. Guigue P. Bonifazi P. Batista-Brito R. Allene C. Ribas A. (2011). Pioneer GABA cells comprise a subpopulation of hub neurons in the developing hippocampus. Neuron 71, 695–709 10.1016/j.neuron.2011.06.018 21867885 PMC3163067

[B65] Picken Bahrey H. L. Moody W. J. (2003). Early development of voltage-gated ion currents and firing properties in neurons of the mouse cerebral cortex. J. Neurophysiol. 89, 1761–1773 10.1152/jn.00972.2002 12611962

[B66] Radnikow G. Feldmeyer D. Lubke J. (2002). Axonal projection, input and output synapses and synaptic physiology of Cajal-Retzius cells in the developing rat neocortex. J. Neurosci. 22, 6908–6919 12177189 10.1523/JNEUROSCI.22-16-06908.2002 PMC6757901

[B67] Rheims S. Minlebaev M. Ivanov A. Represa A. Khazipov R. Holmes G. L. (2008). Excitatory GABA in rodent developing neocortex in vitro. J. Neurophysiol. 100, 609–619 10.1152/jn.90402.2008 18497364

[B68] Rivera C. Voipio J. Payne J.A. Ruusuvuori E. Lahtinen H. Lamsa K. (1999). The K^+^/Cl^-^ co-transporter KCC2 renders GABA hyperpolarizing during neuronal maturation. Nature 397, 251–255 10.1038/16697 9930699

[B69] Saransaari P. Oja S. S. (1998). Mechanisms of ischemia-induced taurine release in mouse hippocampal slices. Brain Res. 807, 118–124 10.1016/s0006-8993(98)00793-8 9757014

[B70] Sernagor E. Chabrol F. Bony G. Cancedda L. (2010). GABAergic control of neurite outgrowth and remodeling during development and adult neurogenesis: general rules and differences in diverse systems. Front. Cell. Neurosci. 4:11 10.3389/fncel.2010.00011 20428495 PMC2859806

[B71] Sipila S. T. Huttu K. Soltesz I. Voipio J. Kaila K. (2005). Depolarizing GABA acts on intrinsically bursting pyramidal neurons to drive giant depolarizing potentials in the immature hippocampus. J. Neurosci. 25, 5280–5289 10.1523/jneurosci.0378-05.2005 15930375 PMC6725004

[B72] Spitzer N. C. (2006). Electrical activity in early neuronal development. Nature 444, 707–712 10.1038/nature05300 17151658

[B73] Stell B. M. Mody I. (2002). Receptors with different affinities mediate phasic and tonic GABA(A) conductances in hippocampal neurons. J. Neurosci. 22, RC223 (1–5) 12006605 10.1523/JNEUROSCI.22-10-j0003.2002 PMC6757628

[B74] Sturman J. A. Lu P. Xu Y. X. Imaki H. (1994). Feline maternal taurine deficiency: effects on visual cortex of the offspring. A morphometric and immunohistochemical study. Adv. Exp. Med. Biol. 359, 369–384 10.1007/978-1-4899-1471-2_38 7887277

[B75] Tamamaki N. Yanagawa Y. Tomioka R. Miyazaki J. Obata K. Kaneko T. (2003). Green fluorescent protein expression and colocalization with calretinin, parvalbumin and somatostatin in the GAD67-GFP knock-in mouse. J. Comp. Neurol. 467, 60–79 10.1002/cne.10905 14574680

[B76] Van Eden C. G. Parmar R. Lichtensteiger W. Schlumpf M. (1995). Laminar distribution of GABA_A_ receptor α_1_, β_2_ and γ_2_ subunit mRNAs in the granular and agranular frontal cortex of the rat during pre- and postnatal development. Cereb. Cortex 5, 234–246 10.1093/cercor/5.3.234 7613079

[B77] Wang D. D. Kriegstein A. R. (2008). GABA regulates excitatory synapse formation in the neocortex via NMDA receptor activation. J. Neurosci. 28, 5547–5558 10.1523/jneurosci.5599-07.2008 18495889 PMC2684685

[B78] Wang D. D. Kriegstein A. R. (2009). Defining the role of GABA in cortical development. J. Physiol. 587, 1873–1879 10.1113/jphysiol.2008.167635 19153158 PMC2689328

[B79] Wojcik S. M. Katsurabayashi S. Guillemin I. Friauf E. Rosenmund C. Brose N. (2006). A shared vesicular carrier allows synaptic corelease of GABA and glycine. Neuron 50, 575–587 10.1016/j.neuron.2006.04.016 16701208

